# Research on Microstructure and Properties of AlSi10Mg Fabricated by Selective Laser Melting

**DOI:** 10.3390/ma15072528

**Published:** 2022-03-30

**Authors:** Wei Pan, Zhanggen Ye, Yongzhong Zhang, Yantao Liu, Bo Liang, Ziyu Zhai

**Affiliations:** 1 National Engineering & Technology Research Center for Non-Ferrous Metals Composites, GRINM Group Corporation Limited, Beijing 101407, China; panweiyzzzl@163.com (W.P.); liuyantaobj@163.com (Y.L.); byliangbo@126.com (B.L.); summer9199@126.com (Z.Z.); 2 GRINM Metal Composites Technology Co., Ltd., Beijing 101407, China; 3 General Research Institute for Nonferrous Metals, Beijing 100088, China

**Keywords:** selective laser melting, aluminum alloy, AlSi10Mg, microstructure, mechanical properties

## Abstract

In order to obtain high-performance aluminum alloy parts fabricated by selective laser melting, this paper investigates the relationship between the process parameters and microstructure properties of AlSi10Mg. The appropriate process parameters are obtained: the layer thickness is 0.03 mm, the laser power is 370 W, the scanning speed is 1454 mm/s, and the hatch spacing is 0.16 mm. With these process parameters, the ultimate tensile strength of the as-printed status is 500.7 ± 0.8 MPa, the yield strength is 311.5 ± 5.9 MPa, the elongation is 7.7 ± 0.5%, and the relative density is 99.94%. After annealing treatment at 275 °C for 2 h, the ultimate tensile strength is 310.8 ± 1.3 MPa, the yield strength is 198.0 ± 2.0 MPa, and the elongation is 13.7 ± 0.6%. The mechanical properties are mainly due to the high relative density, supersaturate solid solution, and fine dispersed Si. The supersaturate solid solution and nano-sized Si formed by the high cooling rate of SLM. After annealing treatment, the Si have been granulated and grown significantly. The ultimate tensile strength and yield strength are reduced, and the elongation is significantly improved.

## 1. Introduction

Selective laser melting (SLM) is a type of additive manufacturing. Different from the traditional machining method of subtractive manufacturing, additive manufacturing is an incremental interlayer cumulative manufacturing. The main metal additive manufacturing technologies include selective laser melting, electron beam melting forming, and laser cladding forming [1,2,3].

SLM is a rapid manufacturing method directly from model to part [4,5]. Compared with traditional manufacturing technology, the SLM can directly manufacture complex shapes metal parts without a mold [6]. It has the advantages of high material utilization and a short processing cycle. The parts can be directly used after heat treatment and polishing, or other surface treatments. Due to the characteristics and advantages of SLM, it has been attracted widespread attention [7,8] and SLM has been developed rapidly in the past 10 years. At present, Ti-based alloy [9,10], Fe-based alloy [11,12,13], Ni-based alloy [14,15,16] manufactured by SLM has been more researched and gradually moving towards application. Therefore, SLM is also expected to provide a new rapid manufacturing method for aluminum alloy.

As a kind of traditional metal material, aluminum alloy has been widely used in aviation, aerospace, automobile, machinery, and other industries, as it has high specific strength, high specific rigidity, and corrosion resistance [17,18]. At present, more and more lightweight design is considered in the parts. The traditional processing methods such as casting, forging, extrusion, and rolling are time-consuming, laborious, and even powerless when these methods are used to manufacture the complex and irregular-shaped parts [19,20]. SLM has great advantages in manufacturing complex and irregular parts. However, aluminum alloy manufactured by SLM has some difficulty due to the poor powder fluidity, high reflectivity, and high thermal conductivity [21,22].

At present, the aluminum alloys fabricated by SLM are mainly Al-Si [23,24], Al-Mg [25,26], and Al-Mn [27]. For AlSi10Mg fabricated by SLM, Li et al. [28] studied the effects of different construction angles on the microstructure and properties. The mechanical properties have been optimized. And the 60° sample of as-processed status had a good strength with a tensile strength of 463.54 MPa, and yield strength of 283.37 MPa. Amir et al. [29] proposed the effect of the stress relief treatment and heated build platform on the mechanical properties and microstructure. It was found that with increasing distance from the heated build platform, there was a graded increase in the surface hardness and dynamic performance, and the relative density of as-printed status is 98.71 ± 0.3%. Wu et al. [30] determined that the influence of the volume energy density on the keyhole formation, microstructural, and mechanical properties, and the three melting modes could be distinguished during the laser melting process. The best relative density is 97.1 ± 0.05% when the volume energy density is 37 J/mm^3^. Maamoun et al. [31] studied the influence of SLM process parameters on the quality of as-built AlSi10Mg parts according to the mutual connection between the microstructure characteristics and mechanical properties. The optimized parameters were investigated to eliminate internal microstructure defects. The ultimate tensile strength of the optimized performance is 396.5 MPa, and the yield strength is 196 MPa.

In summary, the mechanical properties of AlSi10Mg fabricated by SLM are not good enough in the existing research, and the analysis of its forming and strengthening mechanism is not sufficient. In order to study the influence of process parameters on the microstructure and properties, and to analyze the strengthening mechanism, this paper used SLM combined with analyzing various properties of powder, process parameters optimized, and microstructure. The effects of different volume energy density and annealing treatment on the microstructure and properties were investigated, and the strengthening mechanism was analyzed. The microstructure evolution was observed by optical microscope (OM), scanning electron microscope (SEM), and transmission electron microscope (TEM). The internal defects were analyzed by computed tomography (CT). The mechanical properties were studied by a static tensile test at room temperature.

## 2. Materials and Methods

### 2.1. Materials

In this study, AlSi10Mg alloy gas atomized powder was used as feedstock material, fabricated by General Research Institute for Nonferrous Metals (Beijing, China). The chemical composition of the Si, Mg, and O was separately measured by photometry, ICP-AES and inert gas pulse infrared absorption thermal conductivity (Table 1). The air classifier was used to screen AlSi10Mg powder. EP-M250 metal 3D printer (Beijing Eplus3D Technology Co., Ltd., Beijing, China) was used.

**Figure 1:**
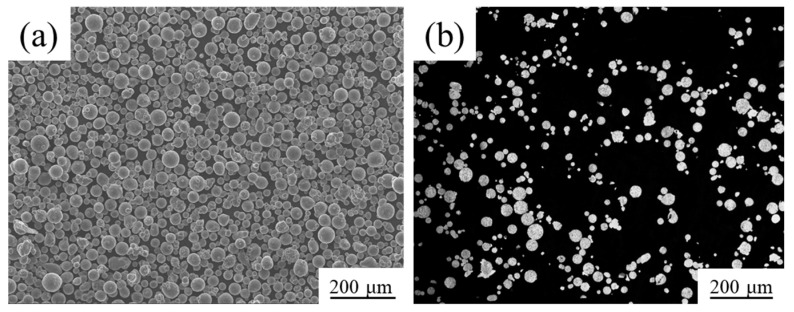
The morphology and cross-sectional microstructure of AlSi10Mg powder: (**a**) powder morphology; (**b**) cross-sectional microstructure.

**Figure 2:**
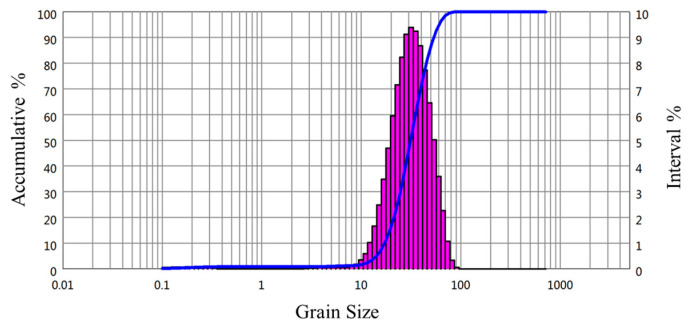
Particle size distribution of AlSi10Mg alloy powder.

The powder morphology observed by JSM-7900F field emission scanning electron microscope (JEOL, Akishima, Tokyo, Japan), is shown in Figure 1a. It can be observed that most of the powder was spherical or quasi-spherical. A small quantity of powder particles presented irregular shapes, and there was a small amount of mutual adhesion between the powder particles. The reason for the mutual adhesion is that the incompletely solidified liquid droplets collided with the solidified powder during the gas atomization. The flowability of powder is fine, and it can ensure uniformity and stability during the powder laying. Figure 1b shows the cross-sectional microstructure observed by Zeiss Axiovert 200 MAT optical microscope (Carl Zeiss AG, Oberkochen, Baden-Württemberg, Germany). It can be seen that there are very few hollow powders, and it was beneficial to increase the relative density of the sample. The particle size distribution was tested by a laser particle size analyzer, as shown in Figure 2. The diameter of the 10% volume fraction powder was less than 17.23 μm (D10 = 17.23 μm), the D50 was 31.43 μm, and the D90 was 53.30 μm. The particle size was mainly in the range of 15–53 μm, which will help to ensure the smoothness and stability during the powder laying.

### 2.2. Forming Process, Microstructure, and Mechanical Properties Analysis

**Figure 3:**
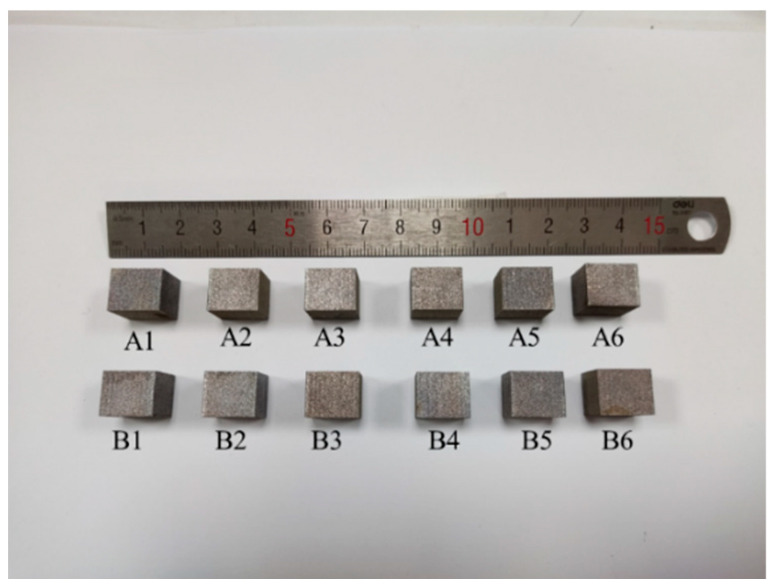
Specimens fabricated by SLM with different processing parameters.

The EP-M250 metal 3D printer (Beijing Eplus3D Technology Co., Ltd., Beijing, China) is equipped with a fiber laser (IPG, Burbach, North Rhine-Westphalia, Germany). The rated power of the laser is 500 W. Before SLM processing, it was necessary to use software (Materialise Magics,21.0, Materialise, Leuven, Belgium) to mark and slice the 3D digital model and use EPHatch to set process parameter. The relationship between the volume energy density (ED_v_) and the various process parameters is defined as follows:

ED_v_ = p/(hvd) (1)

where p is the laser power (W), v is the laser scanning speed (mm/s), d is the hatch spacing (mm), and h is the slice thickness of the powder (mm). In this study, the laser power was rarely used more than 400 W to ensure the stability of the laser output according to the manufacturer’s recommendations. The particle size of this powder is mainly in the range of 15–53 μm, so it is difficult to set the layer thickness significantly less than 0.03 mm. According to previous experiments, the laser power (370 W) and layer thickness (0.03 mm) are appropriate. Therefore, the layer thickness (0.03 mm) and laser power (370 W) were kept as constants. The volume energy density ED_v_ was 49–59 J/mm^3^, the hatch spacing d was 0.16–0.18 mm, and the scanning speed v was 1161–1537 mm/s based on the formula (1). The detailed processing parameters are shown in Table 2. It was necessary to fill the forming chamber with Ar, and strictly control the oxygen content below 100 ppm to prevent oxidation of aluminum alloy during SLM processing. During printing, the laser was stripe-scanned; stripe width was 10 mm (Figure 3 shows the sample). The surface of the sample was smooth and bright. No cracks, deformation, and spheroidization were observed on the surface. This means that AlSi10Mg powder had good processability with these processing parameters.

**Figure 4:**
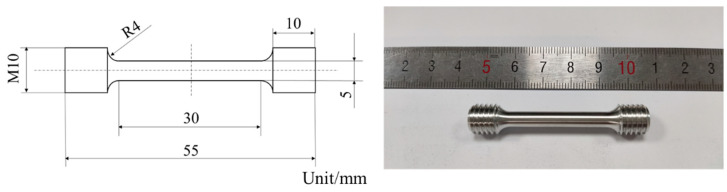
Schematic and physical diagram of the dog-bone rod-shaped standard tensile specimen.

After SLM processing, the samples were cut along the direction perpendicular to the baseplate. The samples were polished with metallographic emery paper, and polished to a mirror-like surface by a polisher. The density was measured by the Archimedes Method (a theoretical density ~2.68 g/cm^3^ was used). The specimens were etched with Keller’s etchant (95% H_2_O, 2.5% HNO_3_, 1.5% HCL, 1.0% HF) (GRINM Metal Composites Technology Co., Ltd., Beijing, China), and finally the sample was rinsed with distilled water. The samples were observed by Zeiss Axiovert 200 MAT optical microscope (Carl Zeiss AG, Oberkochen, Baden-Württemberg, Germany), JSM-7900F field emission scanning electron microscope (JEOL, Akishima, Tokyo, Japan) and FEI Tecnai F20 transmission electron microscope (FEI, Hillsboro, Oregon, United States). According to the analysis of metallographic microstructure and density, the optimal process parameters were selected to fabricate the tensile rods. The tensile rods were processed in the direction of perpendicular to the baseplate. The schematic and physical diagrams of dog-bone rod-shaped standard tensile specimens are shown in Figure 4. The static tensile test at room temperature was carried out by the Quasar10 tensile testing machine, according to the GB/T 228.1-2010 test standard. In order to reduce the error of mechanical properties, three tensile specimens of each process parameter were tested, and the arithmetic mean of the mechanical properties was calculated. Phase constitution of the samples was studied by an X-ray diffractometer (XRD, SmartLab 9 KW) (Rigaku, Akishima, Tokyo, Japan) with Cu-Kα1 radiation at 40 kV and 150 mA. Finally, the appropriate process parameters and specimens were selected by comparing the tensile property. The annealing was carried out at 275 °C for 2 h. And the changes of properties and microstructure between as-printed status and annealing status were analyzed. The defect of the as-processed sample was tested by computer tomography (CT, V|tome|xs240/180, GE Sensing & Inspection Technologies GmbH, Frankfurt, Hesse-Darmstadt, Germany), and the resolution was 6 μm.

## 3. Results

### 3.1. Process Optimization

**Figure 5:**
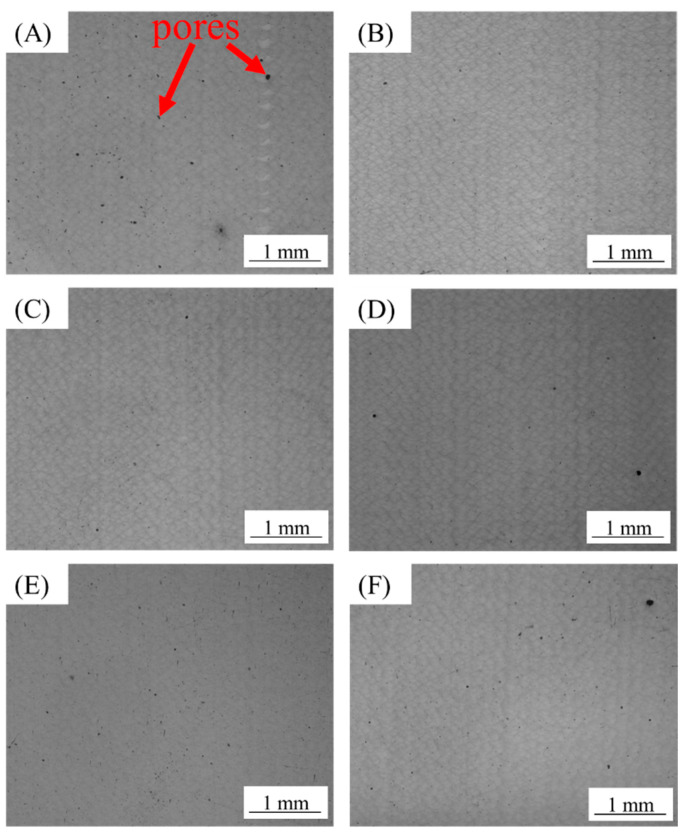
Optical micrographs of AlSi10Mg fabricated with hatch spacing of 0.16 mm and at different volume energy density, showing variation in porosity: (**A**) Process A1; (**B**) Process A2; (**C**) Process A3; (**D**) Process A4; (**E**) Process A5; (**F**) Process A6.

**Figure 6:**
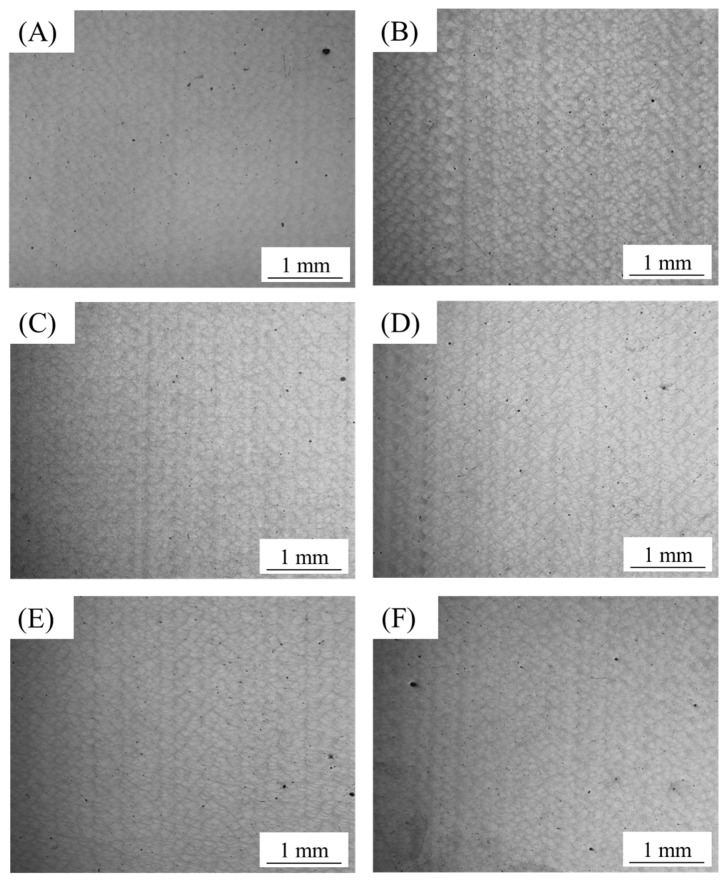
Optical micrographs of AlSi10Mg fabricated with hatch spacing of 0.18 mm and at different volume energy density, showing variation in porosity: (**A**) Process B1; (**B**) Process B2; (**C**) Process B3; (**D**) Process B4; (**E**) Process B5; (**F**) Process B6.

In this study, the detailed process parameters are shown in Table 2. Figure 5 and Figure 6 are the metallographic photos of the samples.

**Figure 7:**
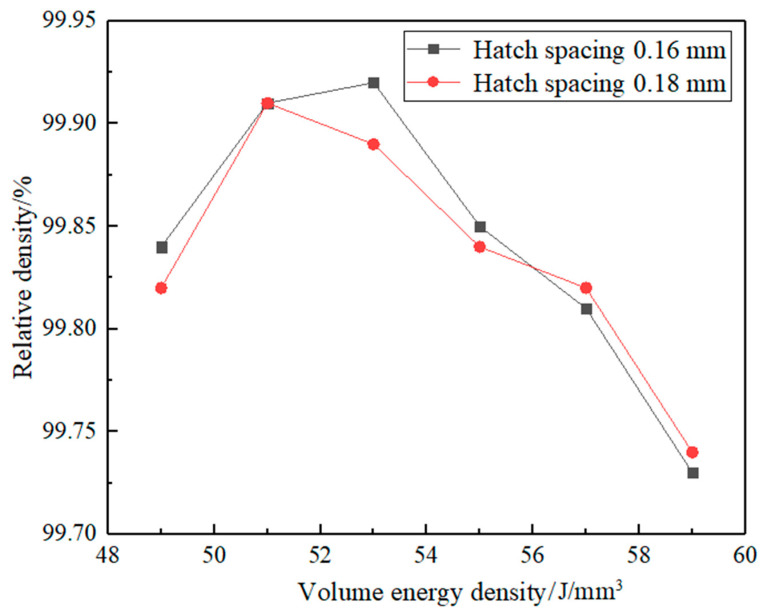
Relative density of AlSi10Mg fabricated with hatch spacing of 0.16 and 0.18 mm, and at different volume energy density/scanning speeds.

As shown in Figure 5 and Figure 6, there were no cracks in each sample. This indicates that AlSi10Mg alloy powder had good formability with these process parameters. There is a certain amount of pore in each sample. Figure 7 shows the relative density of the sample with hatch spacing of 0.16 and 0.18 mm. As shown above, A2, A3, B2, and B3 had fewer pores and higher density.

### 3.2. Microstructure and Mechanical Properties

**Figure 8:**
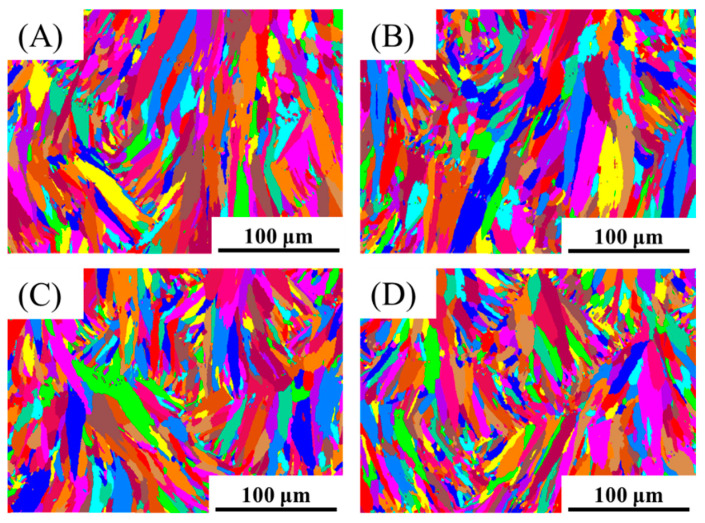
EBSD images of the samples in the as-printed status: (**A**) Process A2; (**B**) Process A3; (**C**) Process B2; (**D**) Process B3.

**Figure 9:**
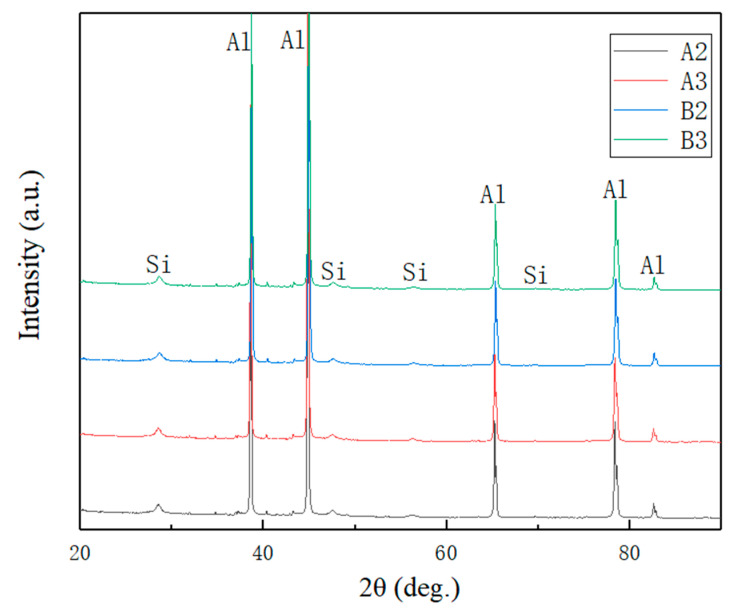
XRD patterns of the A2, A3, B2, and B3 samples in the as-printed status.

**Figure 10:**
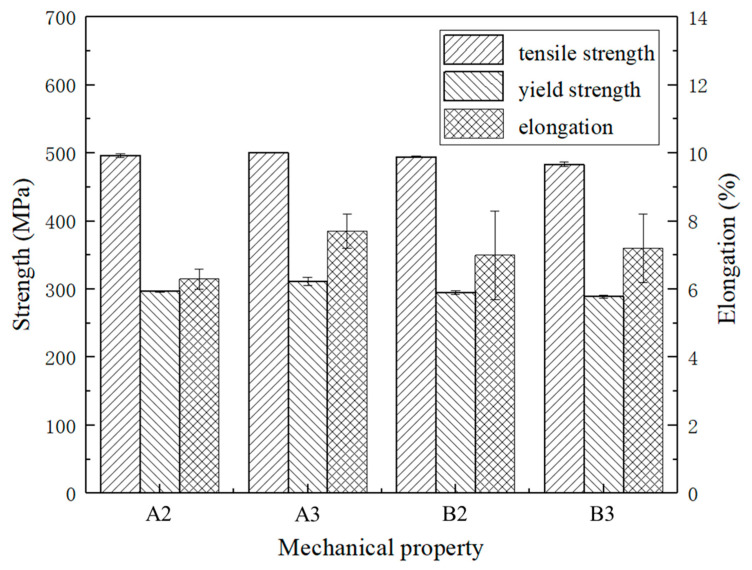
Tensile mechanical properties of A2, A3, B2, and B3 samples in the as-printed status.

Figure 8 shows the EBSD images of A2, A3, B2, and B3 specimens. There are analogous microstructure and grain size distributions. Figure 9 shows the XRD patterns of the A2, A3, B2, and B3 samples in the as-printed status. As shown in Table 3, these are the tensile mechanical properties of the samples A2, A3, B2 and B3. Figure 10 shows the corresponding histogram; the mechanical properties of each sample are comparable and stable.

**Figure 11:**
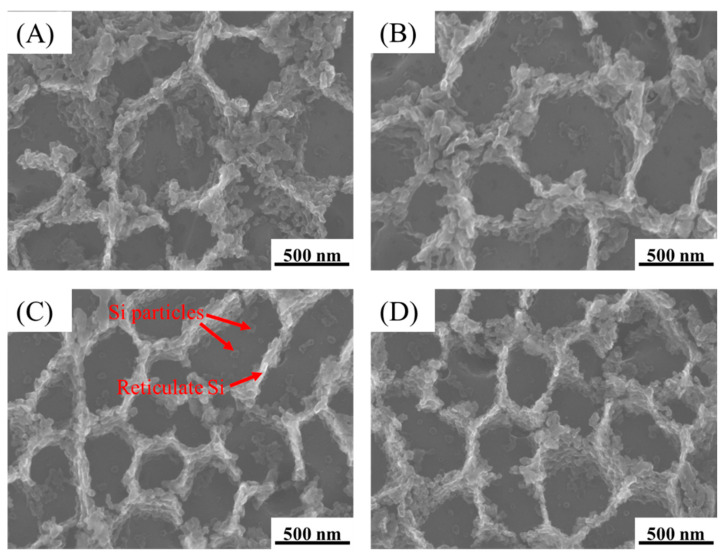
The microstructure morphology of the samples treated by deep-etching: (**A**) Process A2; (**B**) Process A3; (**C**) Process B2; (**D**) Process B3.

**Figure 12:**
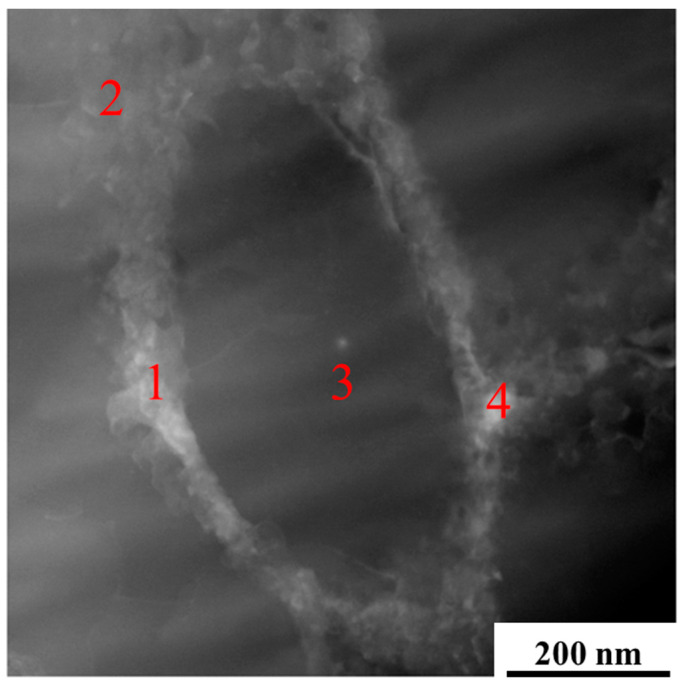
TEM photo of A3 sample in as-printed status, 1–4 are different positions of the photo.

Figure 11 shows the micromorphology of A2, A3, B2, and B3 treated by deep-etching. Based on Figure 9 and Figure 12 and Table 4, the microstructure of A2, A3, B2, and B3 was mainly composed of aluminum matrix, dispersively distributed Si particles of a few tens of nanometers, and reticulate Si with a thickness of one or two hundred nanometers. There was little difference between each sample.

As shown above, the microstructure and mechanical properties of A2, A3, B2, and B3 samples were approximate. The sample A3 was selected for further analysis.

**Figure 13:**
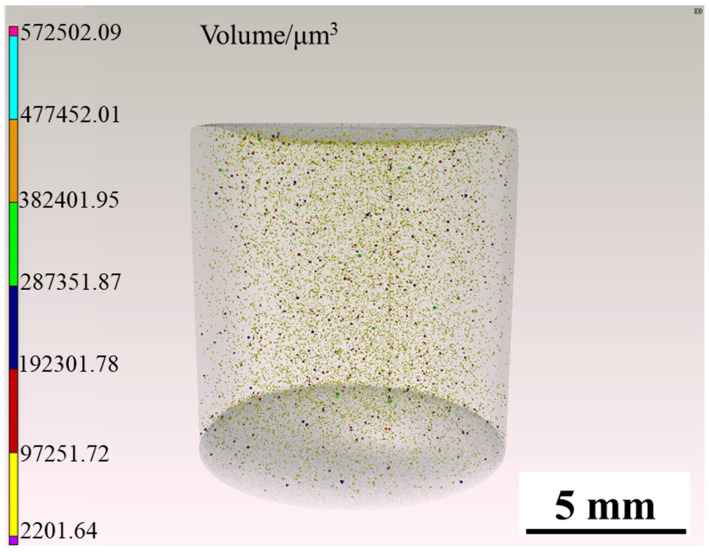
Three-dimensional reconstruction of the A3 sample tested by CT.

Figure 13 is the three-dimensional internal defects reconstruction of the sample A3 tested by CT. It can be seen that there was no crack inside the sample, but there is a certain amount of small pores, and the relative density was 99.94%.

**Figure 14:**
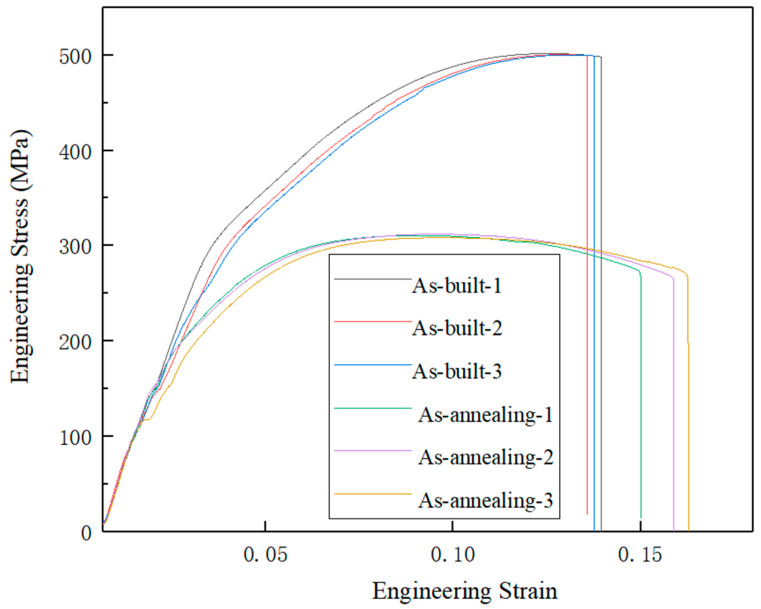
Engineering stress–strain curves of the AlSi10Mg alloys in the as-built state and after annealing treatment at 275 °C for 2 h.

Table 5 shows the mechanical properties of the samples A3 after annealing treatment at 275 °C for 2 h and as-printed status. Figure 14 shows the engineering stress–strain curves of the AlSi10Mg alloys in the as-built state and after annealing treatment at 275 °C for 2 h. After annealing treatment, the ultimate tensile strength and yield strength are reduced, and the elongation is significantly improved.

## 4. Discussion

### 4.1. Influence of Process Parameters on the Microstructure and Mechanical Properties

During the SLM processing, the process parameters are the key influencing factors of the microstructure and performance [32]. According to the previous experiments, this work studied the influence of energy density on the microstructure and mechanical properties under different hatch spacing.

As shown in Figure 5 and Figure 7, when the scanning distance was 0.16 mm, the pores of A2 and A3 samples were decreased compared with the A1 sample. But the number of pores gradually increased after A3. The reason is that the interaction time of laser and powder is longer when the scanning speed is reduced within a relatively small volume energy density. And the powder will absorb enough energy, which will result in a relatively high temperature of the molten pool and a longer existence time. Then, it will result in relatively low melt viscosity and surface tension, which is conducive to the smooth flow of gas in the molten pool. Therefore, the gas between powder particles can escape sufficiently to reduce pores and improve relative density. When the volume energy density increases continuously, the interaction time between the laser and powder will be too long. The powders will absorb too much energy, causing the molten pool temperature to be too high and the existence time of the molten pool to be too long. The laser will have a great impact on the molten pool. Then larger pores will be formed [33,34]. Therefore, when the volume energy density increases continuously, the pores will increase. And the relative density will decrease. Thus, A2 and A3 samples have the least pores. According to Figure 6 and Figure 7, when the scanning distance was 0.18 mm, the pores firstly decreased and then increased with the volume energy density increased, and the pores of B2 and B3 were the least. There is an analogous change rule of the relative density when the laser scanning distance is 0.16 mm and 0.18 mm.

For the AlSi10Mg manufactured by SLM, the pores are the main defect. The reason is that the stress concentration will be formed around these pores when the specimens are static tensile tested and the yield limit is first reached. Then, the cracks will be produced, which will cause rapid crack propagation and ultimately lead to the fracture of the specimens.

Table 3 shows the tensile mechanical properties of A2, A3, B2, and B3 samples. And according to Figure 5, Figure 6 and Figure 7, it can be seen that the microstructure of these samples is dense. The mechanical properties are little different and stable. Among them, the A3 sample has the best strength. The ultimate tensile strength of the as-processed A3 is 500.7 ± 0.8 MPa, the yield strength is 311.5 ± 5.9 MPa, and the elongation is 7.7 ± 0.5%. Compared with the existing studies of SLM [28,31], the mechanical properties have been obviously improved. Compared with the as-cast mechanical properties of die casting AlSi10Mg after process optimization (ultimate tensile strength is 353.31 MPa, the yield strength is 182.97 MPa, and the elongation is 3.478%) [35], the mechanical properties of AlSi10Mg manufactured by SLM have been improved a lot.

**Figure 15:**
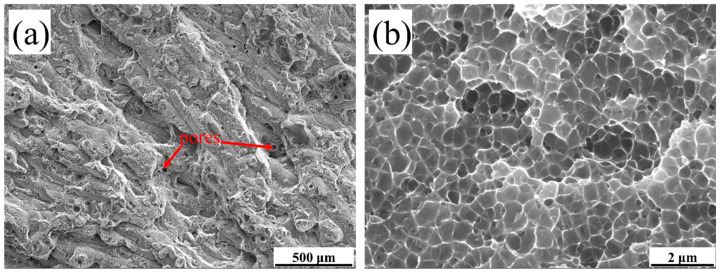
SEM photos of tensile fracture surface of the A3 in as-processed status: (**a**) ×50; (**b**) ×10,000.

Figure 15 shows the fracture surface of the A3 tensile sample. The fracture forms of the as-processed status are ductile fracture. The small amount of pore in Figure 15a was caused by the gas between the powder wrapped in the molten pool and rapid melting and cooling of the molten pool. The pores correspond to the gas holes of Figure 5 and Figure 6. The pores are the weak areas of sample. As shown in Figure 15b, the fracture surface at high magnification is similar to the sub-micron dimple-like structure.

According to Figure 9, Figure 11, and Figure 12 and Table 4, the phases composition of the sample is only aluminum and silicon, and the microstructure is composed of aluminum matrix, dispersively distributed nano-scale Si particles and reticulate Si. Mg_2_Si is not observed. The reason is that the formation and growth of Si particles and Mg_2_Si mainly depend on the Si and Mg element diffusion; the cooling rate of SLM is much higher than traditional processing, which greatly limits the diffusion of Si and Mg elements. Therefore, the rapid solidification of the molten pool leads to that diffusion of elements being limited, and the supersaturate solid solution, the small size, and uniformly distributed nano-scale Si particles are obtained [36]. The supersaturate solid solution (solid solution strengthening) fine and uniformly distributed Si particles (second phase strengthening) are beneficial to improve the mechanical properties of AlSi10Mg.

According to Figure 13, the relative density of the sample A3 is 99.94%. Compared with other studies [29,30], the relative density has been significantly improved. There are no cracks in the sample, and a small amount of small pores are dispersively distributed.

No crack, high relative density, supersaturate solid solution, fine and dispersively distributed Si particles determine that the sample has excellent mechanical properties.

### 4.2. Effect of Annealing Treatment on the Microstructure and Mechanical Properties

Because of the high-energy laser and fast scanning of the laser, a large temperature gradient is generated between the molten pool and substrate. The large temperature gradient will lead to non-uniform thermal expansion, which will result in residual stress within the sample [34]. Residual stress will affect the dimensional accuracy of the samples, and even lead to warp, crack, or the interruption of SLM processing. Therefore, as-processed samples need to be annealed to reduce the residual stress and improve the plastic toughness at the same time.

**Figure 16:**
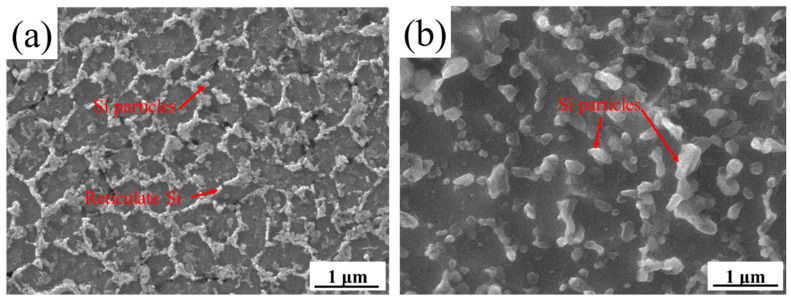
The microstructure photo of A3 sample after deep etching, (**a**) as-printed status, (**b**) annealing status at 275 °C/2 h.

Table 5 shows the mechanical properties of the A3 sample in as-processed status and annealed at 275 °C for 2 h. After annealing, the ultimate tensile strength is 310.8 ± 1.3 MPa, the yield strength is 198.0 ± 2.0 MPa and the elongation is 13.7 ± 0.6%. The ultimate tensile strength and yield strength are reduced, and the elongation is significantly improved. The mechanical properties are also stable as the sample is in as-printed status. Figure 16 shows the micromorphology by deep corrosion of the A3 sample in annealing status. Compared with the as-printed status, the Si have been granulated and grown significantly after annealing, and the number of Si particles is reduced. The growth of Si particles and the decrease of solid solubility will reduce the strengthening effect, which will reduce the material strength [37].

## 5. Conclusions

In this experiment, the relationship between the process parameters and microstructure, mechanical properties of the AlSi10Mg were studied. The strengthening mechanism of AlSi10Mg fabricated by SLM was analyzed, as well as the microstructure and mechanical properties of samples in as-printed status and after annealing treatment. The conclusions drawn from the experimental observation are as follows:

1. When the layer thickness, laser power, and hatch spacing remained unchanged, the relative density will increase first, and then decrease with the increase of the volume energy density. The reason is when the volume energy density is relatively low, the viscosity and surface tension of the molten pool will decrease with the increase of the volume energy density. It is beneficial to the escape of the gas in the molten pool, and this improves the relative density. When the volume energy density increases continuously, the interaction time between laser and powder will be too long. Then the laser will have a strong impact on the molten pool, and form large pores, which will lead to the decrease of relative density.
2. When the layer thickness is 0.03 mm, the laser power is 370 W, the laser scanning speed is 1454 mm/s, and the hatch spacing is 0.16 mm, the mechanical properties of the as-printed status is excellent and stable. The ultimate tensile strength of the as-printed status is 500.7 ± 0.8 MPa, the yield strength is 311.5 ± 5.9 MPa, the elongation is 7.7 ± 0.5%, and the relative density is 99.94%. After annealing treatment at 275 °C for 2 h, the ultimate tensile strength is 310.8 ± 1.3 MPa, the yield strength is 198.0 ± 2.0 MPa, and the elongation is 13.7 ± 0.6%. The ultimate tensile strength and yield strength are reduced due to the growth of Si particles and the decrease of solid solubility. And the elongation is significantly improved.
3. The mechanical properties of AlSi10Mg are mainly due to the supersaturate solid solution (solid solution strengthening), the high relative density, and finely dispersed Si (second phase strengthening). The supersaturate solid solution and submicron Si are generated by the high cooling rate. Because the high cooling rate can restrict the diffusion of elements during the solidification. After annealing treatment, the Si will be granulated and grow significantly, and solid solubility will decrease.

These results analyze the strengthening mechanism, which will help to optimize the microstructure and mechanical properties of AlSi10Mg fabricated by SLM and provide methods and process parameters to manufacture high-performance AlSi10Mg alloy parts.

## Figures and Tables

**Table 1:** Composition of AlSi10Mg powder (wt.%).

Elements	Si	Mg	O	Al
wt.%	10.11	0.28	0.044	Bal.

**Table 2:** Processing parameters of SLM.

No.	Hatch Spacing (d)/mm	Laser Power (P)/w	Volume Energy Density (ED_v_) J/mm^3^	Layer Thickness (h)/mm	Scan Speed (V)/mm/s
A1	0.16	370	49	0.03	1573
A2	0.16	370	51	0.03	1511
A3	0.16	370	53	0.03	1454
A4	0.16	370	55	0.03	1402
A5	0.16	370	57	0.03	1352
A6	0.16	370	59	0.03	1306
B1	0.18	370	49	0.03	1398
B2	0.18	370	51	0.03	1344
B3	0.18	370	53	0.03	1293
B4	0.18	370	55	0.03	1246
B5	0.18	370	57	0.03	1202
B6	0.18	370	59	0.03	1161

**Table 3:** Tensile mechanical properties of A2, A3, B2, and B3 samples in the as-printed status.

No.	Ultimate Tensile Strength/MPa	Yield Strength/MPa	Elongation/%
A2	496.0 ± 2.6	297.0 ± 1.0	6.3 ± 0.3
A3	500.7 ± 0.8	311.5 ± 5.9	7.7 ± 0.5
B2	494.3 ± 1.2	294.7 ± 2.5	7.0 ± 1.3
B3	483.3 ± 3.1	289.3 ± 2.1	7.2 ± 1.0

**Table 4:** The EDS analysis of A3 sample in Figure 12 (wt.%).

Destination	Al	Si	Mg
1	60.03	38.12	1.85
2	80.09	19.04	0.87
3	97.30	2.58	0.12
4	46.80	50.51	2.69

**Table 5:** Mechanical properties of A3 in annealing status and as-processed status.

No.	Ultimate Tensile Strength/MPa	Yield Strength/MPa	Elongation/%
A3 in as-processed status	500.7 ± 0.8	311.5 ± 5.9	7.7 ± 0.5
A3 in annealing status	310.8 ± 1.3	198.0 ± 2.0	13.7 ± 0.6

## Data Availability

Date is contained within the article.

## References

[1] Suzuki A., Miyasaka T., Takata N., Kobashi M., Kato M., Kato M. (2021). Control of microstructural characteristics and mechanical properties of AlSi12 alloy by processing conditions of laser powder bed fusion. Addit. Manuf..

[2] Khrapov D., Kozadayeva M., Manabaev K., Panin A., Sjöström W., Koptyug A., Mishurova T., Evsevleev S., Meinel D., Bruno G. (2021). Different Approaches for Manufacturing Ti-6Al-4V Alloy with Triply Periodic Minimal Surface Sheet-Based Structures by Electron Beam Melting. Materials.

[3] Wang W., Xu X., Ma R., Xu G., Liu W., Xing F. (2020). The Influence of Heat Treatment Temperature on Microstructures and Mechanical Properties of Titanium Alloy Fabricated by Laser Melting Deposition. Materials.

[4] Lu B.H., Li D.C. (2013). Development of the additive manufacturing (3D printing) technology. Mech. Build. Autom..

[5] Yap C.Y., Chua C.K., Dong Z., Liu Z.H., Sing S.L. (2015). Review of selective laser melting: Materials and applications. Appl. Phys. Rev..

[6] Wang H.M. (2014). Materials’ fundamental issues of laser additive manufacturing or high-performance large metallic components. Acta Aeronaut. Astronaut. Sin..

[7] Yadroitsev I., Thivillon L., Bertrand P., Smurov I. (2007). Strategy of manufacturing components with designed internal structure by selective laser melting of metallic powder. Appl. Surf. Sci..

[8] Olakanmi E.O. (2013). Selective laser sintering/melting (SLS/SLM) of pure Al, Al-Mg, and Al-Si powders: Effect of processing conditions and powder properties. J. Mater. Processing Technol..

[9] Polina M., Dan E., Guy B.H. (2021). Hydrogen trapping in additive manufactured Ti–6Al–4V alloy. Mater. Sci. Eng. A.

[10] Xi L., Ding K., Gu D., Guo S., Cao M., Zhuang J., Lin K., Okulov I., Sarac B., Eckert J. (2021). Interfacial structure and wear properties of selective laser melted Ti/(TiC + TiN) composites with high content of reinforcements. J. Alloy Compd..

[11] Kořínek M., Halama R., Fojtík F., Pagáč M., Krček J., Krzikalla D., Kocich R., Kunčická L. (2020). Monotonic Tension-Torsion Experiments and FE Modeling on Notched Specimens Produced by SLM Technology from SS316L. Materials.

[12] Kale A.B., Alluri P., Singh A.K., Choi S.H. (2021). The deformation and fracture behavior of 316L SS fabricated by SLM under mini V-bending test. Int. J. Mech. Sci..

[13] Yao D., Liu X., Wang J., Fan W., Li M., Fu H., Zhang H., Yang X., Zou Q., An X. (2021). Numerical insights on the spreading of practical 316 L stainless steel powder in SLM additive manufacturing. Powder Technol..

[14] Cao Y., Zhou X., Cong D., Zheng H., Cao Y., Nie Z., Chen Z., Li S., Gao Z., Cai W. (2020). Large tunable elastocaloric effect in additively manufactured Ni–Ti shape memory alloys. Acta Mater..

[15] Behzad F., Bhushan R.B., Federico V., Amirhesam A., Narges S.M. (2020). Study on variations of microstructure and metallurgical properties in various heat-affected zones of SLM fabricated Nickel–Titanium alloy. Mater. Sci. Eng. A.

[16] Xia M., Gu D., Ma C., Chen H., Zhang H. (2018). Microstructure evolution, mechanical response and underlying thermodynamic mechanism of multi-phase strengthening WC/Inconel 718 composites using selective laser melting. J. Alloy Compd..

[17] Valiev R.Z., Murashkin M.Y., Sabirov I. (2014). A nanostructural design to produce high-strength Al alloys with enhanced electrical conductivity. Scr. Mater..

[18] Wang H.Z., Leung D.Y.C., Leung M.K.H., Ni M. (2009). A review on hydrogen production using aluminum and aluminum alloys. Renew. Sustain. Energy Rev..

[19] Spieringsa A.B., Dawsonb K., Kern K., Palm F., Wegener K. (2017). SLM-processed Sc- and Zr- modified Al-Mg alloy: Mechanical properties and microstructural effects of heat treatment. Mater. Sci. Eng. A.

[20] Sercombe T.B., Schaffer G.B. (2003). Rapid manufacturing of aluminum components. Science.

[21] Zhou L., Pan H., Hyer H., Park S., Bai Y., McWilliams B., Cho K., Sohn Y. (2019). Microstructure and tensile property of a novel AlZnMgScZr alloy additively manufactured by gas atomization and laser powder bed fusion. Scr. Mater..

[22] Zhang J., Song B., Wei Q., Bourell D., Shi Y. (2019). A review of selective laser melting of aluminum alloys: Processing, microstructure, property and developing trends. J. Mater. Sci. Technol..

[23] Cai Y., Lu T., Ma G.D., Li W., Pan Y., Ding H. (2021). Effects of geometrical characteristics on defect distributions in alloy components produced by selective laser melting. China Foundry.

[24] Chen Y., Song S., Zhu S., Cui X., Zhao F. (2021). Selective laser remelting of in-situ Al_2_O_3_ particles reinforced AlSi10Mg matrix composite: Densification, microstructure and microhardness. Vacuum.

[25] Gu D., Zhang H., Dai D., Ma C., Zhang H., Li Y., Li S. (2020). Anisotropic corrosion behavior of Sc and Zr modified Al-Mg alloy produced by selective laser melting. Corros. Sci..

[26] Zhang H., Gu D., Dai D., Ma C., Li Y., Peng R., Li S., Liu G., Yang B. (2020). Influence of scanning strategy and parameter on microstructural feature, residual stress and performance of Sc and Zr modified Al–Mg alloy produced by selective laser melting. Mater. Sci. Eng. A.

[27] Bayoumy D., Schliephake D., Dietrich S., Wu X.H., Zhu Y.M., Huang A.J. (2021). Intensive processing optimization for achieving strong and ductile Al-Mn-Mg-Sc-Zr alloy produced by selective laser melting. Mater. Des..

[28] Li X., Yi D., Wu X., Zhang J., Yang X., Zhao Z., Feng Y., Wang J., Bai P., Liu B. (2021). Effect of construction angles on microstructure and mechanical properties of AlSi10Mg alloy fabricated by selective laser melting. J. Alloy Compd..

[29] Amir B., Grinberg E., Gale Y., Sadot O., Samuha S. (2021). Influences of platform heating and post-processing stress relief treatment on the mechanical properties and microstructure of selective-laser-melted AlSi10Mg alloys. Mater. Sci. Eng. A.

[30] Wu H., Ren Y., Ren J., Liang L., Li R., Fang Q., Cai A., Shan Q., Tian Y., Baker I. (2021). Selective laser melted AlSi10Mg alloy under melting mode transition: Microstructure evolution, nanomechanical behaviors and tensile properties. J. Alloy Compd..

[31] Zhou S.Y., Su Y., Wang H., Enz J., Ebel T., Yan M. (2019). The Effect of Selective Laser Melting Process Parameters on the Microstructure and Mechanical Properties of Al6061 and AlSi10Mg Alloys. Materials.

[32] Kotadia H.R., Gibbons G., Das A., Howes P.D. (2021). A Review of Laser Powder Bed Fusion Additive Manufacturing of Aluminium Alloys: Microstructure and Properties. Addit. Manuf..

[33] Wang X.C. (2017). Microstructure and Properties of Selective Laser Melting AlSi10Mg Alloy.

[34] Zhang W.X. (2008). Research on the Key Technologies for Selective Laser Melting Process.

[35] Liang T. (2012). The Research on the Die Casting Process of Hypoeutectic Al-Si Alloy AlSi10Mg.

[36] Li Y.L. (2015). Numerical Investigation on Temperature Field and Stress Field during Selective Laser Melting of AlSi10Mg.

[37] Wang X.J. (2014). Process Parameters and Properties of Selective Laser Melting Al-Si Alloys.

